# A cross-sectional study of quality of life in a cohort of enteral ostomy patients presenting to a tertiary care hospital in a developing country in South Asia

**DOI:** 10.1186/s13104-017-2406-2

**Published:** 2017-01-31

**Authors:** Umesh Jayarajah, Dharmabandhu N. Samarasekera

**Affiliations:** Department of Surgery, Faculty of Medicine, University of Colombo, P.O. Box 271, Kynsey Road, Colombo 8, Western Province Sri Lanka

**Keywords:** Quality of life, Self-efficacy, Enteral ostomy

## Abstract

**Background:**

Enteral ostomy creation affects the quality of life (QOL) of stoma patients significantly. Studying the QOL and its determinants is important as it may help in the early identification of those with poor QOL leading to appropriate intervention. This study was aimed to assess the possible contributory factors of QOL of stoma patients.

**Methods:**

A cross sectional study was conducted among 43 ostomy patients who presented for follow up at a surgical clinic of a tertiary care hospital in Sri Lanka over a period of 1 year. Relevant demographic and ostomy related data were collected using an interviewer administered questionnaire. Stoma quality of life scale (Stoma-QOL) and stoma care self-efficacy scale (SCSE) which are validated questionnaires were used to assess QOL and self-efficacy in managing stoma respectively. Associations were established using independent samples *t* test and Spearman’s correlation.

**Results:**

The median age of the study participants was 47.5 years (range 18–83). The median follow up duration was 38 months (range 6–183). The mean overall QOL score was 53.07 ± SD 12.68. Approximately 70% of the study participants scored less than 60. Higher QOL was associated with female sex, colostomies, comfortable income and satisfactory sexual activity. Significantly lower overall QOL was found in those who reported a significant change in the style of dressing (p < 0.05), those who felt depressed (p < 0.05), and those who had thoughts of self-harm soon after surgery (p < 0.05). There was a significant positive correlation between QOL and self-efficacy (p < 0.01). Those who took longer time to learn to take care of the stoma had lower QOL (p < 0.05).

**Conclusions:**

The overall QOL score was considerably low in our study. The QOL was significantly associated with self-efficacy which indicates the importance of patient education and training during follow up visits to maintain a higher QOL. Furthermore integrating with other non-surgical specialities to address multi-dimensional problems including psychosocial and sexual aspects may be helpful to achieve a better QOL.

## Background

Intestinal ostomy is a surgical procedure that is done to treat several benign and malignant intestinal conditions. Colorectal malignancies, inflammatory bowel disease, polyposis syndromes and trauma are few conditions in which stomas are used. The two most leading causes are colorectal malignancies and inflammatory bowel disease [1]. Furthermore the incidence of colorectal malignancies are on the rise worldwide which is also now seen among the Asian population. The significant increase in morbidity and mortality has made colorectal cancer a major public health concern. Increase in the incidence of colorectal malignancies has significantly contributed to the increase in ostomy creation [2].

In addition, creation of an ostomy has a significant impact on quality of life (QOL) which can present as physical, psychological or social disability. Restriction of physical activities, dietary restrictions and sexual difficulties are some of the physical factors which influence the QOL. The patients also face significant change in the perception of body image, general lifestyle and relationship with families and friends [3, 4].

One of the main aims of treating ostomy patients is to improve the QOL and currently significant emphasis is given to this aspect [5]. However studies on QOL among stoma patients in the South Asian region are very scarce and sometimes not measured using validated instruments. Studying the QOL and its determinants is important in the management of ostomy patients as it may help in the early identification of those with poor QOL leading to appropriate intervention.

## Methods

### Selection of participants

All patients who presented for follow up at a surgical clinic of a tertiary care hospital in Sri Lanka over a period of 1 year were enlisted. All those who were above 18 years, who were living with an enteral ostomy and having a willingness to participate were recruited. Those with other physical or psychological morbidities that could interfere with self-care were excluded from this study. Finally 43 patients were selected to participate in this study.

### Study design and data collection

A descriptive cross sectional study was carried out. The study was conducted at the National Hospital of Sri Lanka, Colombo. The data were collected during clinic visits. Details on demographic characteristics, details of the disease and surgical details were gathered. Validated questionnaires were used to collect data.

Demographic characteristics analysed in the study included, age, sex, marital status, ethnicity, educational level, body mass index, and social and psychological support. Details of occupation, sexual activity, psychological aspects and change in food and clothing habits were also collected. The clinical records were used to gather data on related details on ostomy, disease and the treatment which included type of surgery and complications of ostomy.

Written informed consent for participation in the study was obtained from all the participants.

### Study instruments

The quality of life was measured using Stoma-QOL scale [5]. This consisted of 20 items that covered several areas which were concerns due to stoma, sleep, sexual activity, relationship with family and close friends and social interactions with others other than family and close friends. Participants responded to 20 items on a 4-point Likert scale with numbers referring to 1 = never, 2 = occasionally, 3 = frequently, or 4 = always. The final value was converted to a continuous scale ranging from 0 to 100. These items were found to define a unidimensional variable according to Rasch specifications (Infit MNSQ < 1.3). Internal consistency reliability was highly reliable as Cronbach’s alpha was 0.92. Spearman’s correlation coefficients of scores across times of administration was >0.88 (p < 0.01), indicating a high test–retest reliability. Results of Rasch specifications as well as classical analysis indicate the suitability of the instrument for clinical research [5].

The level of self-efficacy on caring for stoma was measured using stoma care self-efficacy scale (SCSE) [6]. This consisted of 13 items on self-efficacy of managing a stoma. It is designed to assess a general sense of perceived self-efficacy with the aim to assess the coping with day to day difficulties of managing a stoma. The Cronbach’s alpha was 0.94 indicating a highly reliable internal consistency. Patients responded to a 5 point Likert scale numbered as 1 = not being confident at all, 2 = slightly confident, 3 = fairly confident, 4 = highly confident and 5 = extremely confident. High scores indicate higher perception of self-efficacy. The total score is calculated to 100 whereas less than 50 = low self-efficacy and more than 50 = high self-efficacy. A group of experts specialized in General Surgery, Medical-surgical and psychiatric nursing ascertained the content validity. Contents of the tools were tested regarding to the knowledge accuracy, relevance and competence [6].

The questionnaires which were originally in English were translated to local languages and were administered as an interviewer administered questionnaire to minimize discrepancies. The male patients were interviewed by a male investigator and the female patients by a female investigator to collect data on personal information such as sexual function.

### Data analysis

Data were analysed using SPSS 17.0 statistical software (SPSS Inc., USA). Continuous variables were expressed using means ± standard deviations. For inferential analysis, Spearman’s correlations were used to analyse the relationship between continuous demographic variables, self-care, SQOL scores. For independent samples, a *t* test was used to analyse the relationship between continuous variables and SQOL scores. Statistical testing was performed at the 0.05 significance level.

## Results

### Demographic factors

The median age of the study participants was 47.5 years (range 18–83). The median follow up duration was 38 months (range 6–183). The majority of the participants were males (69.5%), and ethnicity were Sinhalese (62.8%), Tamil (20.9%) and Muslim (16.3%). Of the participants 62.8% were unemployed with 67.4% living married (Table 1).

**Table 1:** Demographic characteristics

		Number	
Sex			
Male		30	(69.5%)
Female		13	(30.2%)
Ethnicity			
Sinhala		27	(62.8%)
Tamil		9	(20.9%)
Muslim		7	(16.3%)
Employment status			
Yes		16	(37.2%)
No		27	(62.8%)
Income			
Comfortable income		7	(16.3%)
Enough to meet important needs		22	(51.2%)
Not enough to meet important needs		14	(32.6%)
Marital status			
Unmarried		10	(23.3%)
Married		29	(67.4%)
Widowed		3	(7.0%)
Separated		1	(2.3%)
Education level			
Below grade 11		19	(44.18%)
Grade 11 and above		24	(55.82%)

### Characteristics of ostomy

The majority of the patients had a colostomy (n = 32, 74.4%) and the commonest indications were carcinoma of the lower rectum and anus (n = 16, 37.2%) followed by inflammatory bowel disease (n = 9, 20.9%). Commonest surgery performed was a defunctioning colostomy (n = 19, 44.2%) followed by abdomino-perineal resection (n = 8, 18.6%). Other common surgeries were anterior resection, Hartmann’s procedure and ileal pouch anal anastomosis.

### Complications of ostomy

The majority of the participants experienced complication of stoma at some point (n = 22, 51.2%). Of the participants 32.5% (n = 14) experienced one complication and 18.6% (n = 8) experienced 2 or more complications. The common complications experienced were skin excoriation (n = 10, 23.3%), parastomal hernia (n = 8, 18.6%) and prolapse (n = 8, 18.6%).

### Quality of life (QOL) following ostomy

The mean overall QOL score was 53.07 ± SD 12.68 (range 18–82) (Table 2). The associations were determined between possible determinant factors and QOL scores considering both overall score and its components separately. The overall score was out of 100. The components were scored by determining the mean (minimum possible score 1 and maximum 4). Approximately 70% of the study participants scored less than 60. The scores on concerns about stoma and sleep were comparatively poorer. The highest correlation with the overall QOL score was seen with social sub-scale (r_s_ = 0.906) followed by sleep (r_s_ = 0.851) and concerns due to ostomy (r_s_ = 0.838). In our cohort of patients, the sexual/body image scores (r_s_ = 0.715) had the lowest impact on the overall quality of life. (r_s_ = Spearman’s correlation co-efficient).

**Table 2:** Overall and specific QOL scores

Scores	Mean	Standard deviation
Overall QOL out of 100	53.07	12.68
Concerns due to stoma	2.24	0.80
Sleep	2.54	0.83
Social and family relationships	2.74	0.92
Sexuality and body image	2.71	0.83

Of the demographic factors, there was higher QOL in female patients which was not statistically significant (p = 0.51). There was no association between age, body mass index, ethnicity, and marital status with QOL. Those who had a “comfortable” income and those who are employed had a higher overall QOL which was not statistically significant. However, those who had a “comfortable” income had a significantly higher scores measuring family and social relationships (Table 3).

**Table 3:** Association between QOL and demographic parameters

		N	Mean	Standard deviation	p value
Overall QOL with sex					
Male		30	51.1890	12.79	0.51
Female		13	57.4254	11.75	
Overall QOL with age					
Age <60		31	52.5361	13.37	0.660
Age >60		12	54.4650	11.12	
Overall QOL with ethnicity					
Sinhala		27	53.72	11.82	0.892
Tamil		9	51.37	16.63	
Muslim		7	52.72	12.00	
Overall QOL with employment status					
Employed		16	54.0694	11.61	0.69
Unemployed		27	52.4848	13.45	
Overall QOL with income					
Comfortable income		7	57.2757	6.54	0.46
Other		33	53.3782	13.42	
Family and social relationships scores with income					
Comfortable income		7	3.3810	0.4879	0.027
Other		33	2.7677	1.0257	
Overall QOL with marital status					
Married		29	52.7945	13.60	0.41
Others		14	53.6543	10.97	
Overall QOL with education					
Less than O/L		33	53.12	13.60	0.96
O/L or above		10	52.90	9.58	
Overall QOL with BMI					
High BMI		25	52.2516	11.76	0.53
Low or normal BMI		17	54.7576	14.37	

*O*/*L* ordinary level examinations

Those who were having a loop ostomy had a higher score. Furthermore, there was higher QOL in permanent ostomies compared to temporary ones. Defunctioning ostomies had a higher QOL compared to other types of surgery. However, the difference seen was not statistically significant (Table 4). Patients who underwent rectal surgery and ostomy creation had lower rate of sexual satisfaction (p = 0.414), higher rate of abstinence following ostomy (p = 0.129) and higher rate of impotence (p = 0.019) which was statistically significant, compared to those who had ostomy creation without rectal surgery. There was no significant difference in the overall quality of life in those who underwent rectal surgery. However, the sexual/body image scores were slightly lower in the rectal surgery group, although it was statistically not significant (p = 0.0.689).

**Table 4:** Association between QOL and ostomy related parameters

		N	Mean	Standard deviation	p value
Overall QOL with type: ileostomy vs colostomy					
Ileostomy		11	50.91	17.26	0.519
Colostomy		32	53.81	10.92	
Overall QOL with type loop vs end					
Loop		26	55.19	12.10	0.178
End		17	49.82	13.20	
Overall QOL with surgery type					
Defunctioning ostomy		19	54.17	10.54	0.619
Other		24	52.20	14.31	
Overall QOL with rectal surgery					
Yes		20	53.74	14.53	0.749
No		23	52.48	11.12	
Sexual/body image scores with rectal surgery					
Yes		20	2.65	0.8269	0.689
No		23	2.75	0.8540	
Overall QOL: permanent vs temporary					
Permanent		23	55.46	13.51	0.188
Temporary		20	50.32	11.35	

Of the participants who were sexually active prior to surgery (n = 27), only 33% (n = 9) resumed sexual activity following surgery. Only 8 patients were satisfied with their sexual activity. The higher scores were noted in all components of QOL score in those who had satisfactory sexual life and those with no change in the style of clothing. Similarly, those who had no change in dietary habits had higher scores in most of the components, except for sexual and body image scores. Of these, the difference noted due to change in style of clothing was statistically significant (p = 0.013). Thus, the impact on the overall scores is not only due to the isolated improvement in the relevant sub-scores, but also due to improvement in other unrelated sub-scores (Table 5).

**Table 5:** Association with QOL and problems due to ostomy

		N	Mean	Standard deviation	p value
Association with sexual satisfaction and QOL					
Overall QOL score					
Yes		8	54.56	16.02692	0.566
No		24	51.29	13.05682	
Scores on concerns of stoma					
Yes		8	2.25	0.94281	0.853
No		24	2.1875	0.77757	
Scores on sleep					
Yes		8	2.5417	0.94176	0.783
No		24	2.4444	0.83212	
Scores on family and social relationships					
Yes		8	2.8571	1.00436	0.625
No		24	2.6607	0.96322	
Scores on sexuality/body image					
Yes		8	2.625	0.82496	0.938
No		24	2.5972	0.87906	
Association with change in clothing style and QOL					
Overall QOL score					
No		19	58.3884	11.92004	0.013
Yes		24	48.8675	11.85794	
Scores on concerns of stoma					
No		19	2.4386	0.86818	0.158
Yes		24	2.0903	0.71893	
Scores on sleep					
No		19	2.8421	0.87749	0.033
Yes		24	2.3056	0.71503	
Scores on family and social relationships					
No		19	3.1729	0.74775	0.005
Yes		24	2.3988	0.90741	
Scores on sexuality/body image					
No		19	2.9123	0.87378	0.15
Yes		24	2.5417	0.77903	
Association with change in dietary habits and QOL					
Overall QOL score					
No		16	54.9544	15.16167	0.461
Yes		27	51.9604	11.11946	
Scores on concerns of stoma					
No		16	2.4896	0.83326	0.122
Yes		27	2.0988	0.75427	
Scores on sleep					
No		16	2.6667	1.03994	0.455
Yes		27	2.4691	0.681	
Scores on family and social relationships					
No		16	2.75	0.98492	0.961
Yes		27	2.7354	0.89452	
Scores on sexuality/body image					
No		16	2.6042	1.00531	0.546
Yes		27	2.7654	0.72686	

Those who claimed to be depressed soon after surgery had significantly lower scores in all components of QOL (p < 0.05) (Table 6). Furthermore those who had suicidal thoughts at some point after stoma also had lower overall QOL (p < 0.05) (Table 7). Significant reduction was seen in scores that measured social component which included, relationship with family, friends and making new relationships. Also the scores measuring concerns due to stoma, sleep, sexuality/body image were low. However, the difference noticed was not statistically significant.

**Table 6:** Association between QOL and feeling “depressed” soon after surgery

	Felt depressed soon after surgery	N	Mean	Standard deviation	p value
Overall QOL score	Yes	19	46.7516	11.84774	0.001
	No	22	59.2200	10.31910	
Scores on concerns of stoma	Yes	19	1.9386	0.67862	0.015
	No	22	2.5303	0.79787	
Scores on sleep	Yes	19	2.2456	0.83031	0.015
	No	22	2.8636	0.73217	
Scores on family and social relationships	Yes	19	2.2857	0.83299	0.001
	No	22	3.1753	0.78362	
Scores on sexuality/body image	Yes	19	2.3333	0.80123	0.003
	No	22	3.0606	0.68727

**Table 7:** Association between QOL and “thoughts of suicide/self-harm at some point due to ostomy”

	Thoughts of suicide/self-harm at some point due to ostomy	N	Mean	Standard deviation	p value
Overall QOL score	Yes	5	42.2880	17.45353	0.042
	No	38	54.4937	11.47986	
Scores on concerns of stoma	Yes	5	2.0333	0.87718	0.536
	No	38	2.2719	0.79546	
Scores on sleep	Yes	5	2.0667	0.95452	0.173
	No	38	2.6053	0.80111	
Scores on family and social relationships	Yes	5	1.8571	0.88641	0.020
	No	38	2.8571	0.86674	
Scores on sexuality/body image	Yes	5	2.2000	1.09545	0.151
	No	38	2.7719	0.78692	

The mean self-efficacy score was 70.57 ± SD 14.68. About 50% had scores more than 75. We noted significant correlation between self-efficacy in stoma care and QOL. There was significant positive association with overall score (p = 0.003) and components related to concerns of stoma (p = 0.002), sleep (p = 0.028), and social relationship (p = 0.015) (Table 8). There was a significant negative correlation between time taken to train to care for stoma and scores related to concerns of stoma. This means sooner they train themselves to care for the stoma better the QOL (p = 0.003). There was no correlation between duration of follow up and both QOL and self-efficacy scores.

**Table 8:** Correlation between QOL and self-efficacy and time taken to learn to take care of stoma

	Scores	N	Spearman’s rho	p value
Self-efficacy	Overall QOL	43	0.446	0.003
	Concerns of stoma	43	0.459	0.002
	Sleep	43	0.336	0.028
	Family and other social relationships	43	0.370	0.015
	Sexuality and body image	43	0.214	0.167
Time taken to learn to take care of stoma	Overall QOL	43	−0.277	0.083
	Concerns of stoma	43	−0.462	0.003
	Sleep	43	−0.127	0.435
	Family and other social relationships	43	−0.095	0.558
	Sexuality and body image	43	−0.152	0.348

## Discussion

The results of this study suggest that there is significant impact on the QOL following an ostomy. About 70% of the participants had overall scores less than 60%. Our study is also compatible with other studies which showed similar problems faced by ostomy patients [7–9]. There were comparatively lower scores on components measuring stoma concerns and sleep in our cohort. The scores on sexuality/body image and social scores were comparatively better.

Only 16.3% had a comfortable income in our cohort and 32% did not have an income even to cover their basic needs. Sri Lanka being a developing country, this reflects the poor socioeconomic background the patients are from and this factor may have had an influence on reduced QOL. The overall QOL was not statistically different between the groups. However, the family relationship component had a statistically significant difference and those who had a comfortable income had a higher score. The additional economic burden due to the stoma may cause family problems and relationship issues which may have influenced the scores. These findings are also consistent with other studies which gave similar results [10–12]. Therefore, it is important identify the individuals who are from a poor socioeconomic background and refer them to the social worker before surgery, to help them to find an alternative source of income and socioeconomic support, if they would become unable to continue their job following surgery. Furthermore, it is important to counsel those who will be able to continue their jobs following surgery, to avoid unemployment merely due to social concerns.

The majority of the participants who were sexually active before ostomy did not resume their sexual activity after surgery. Only 33.3% of participants resumed sexual activity. Therefore the findings are also consistent with other similar studies [8, 13]. However, the proportion of patients resuming their sexual activity was considerably low in our population, and also the proportion of those who were satisfied with their sexual activity was also low. This may be due to lack of effective counselling of our patients. Furthermore the patients do not openly discuss these issues with the health care providers due to embarrassment. Therefore, it is essential to counsel the patients prior to surgery, especially those who would undergo rectal surgery, regarding the potential sexual issues and risk of impotence following rectal surgery. Furthermore, relevant patients should be routinely questioned about their sexual wellbeing during follow up assessments and prompt referral should be arranged as needed.

There was no significant difference between QOL and change in dietary habits which may indicate that they are able to successfully cope with the diet change. However, a study done in Netherlands in 2000 showed that there was significant reduction in the QOL in those who were prescribed dietary modifications [4]. Change in the style of clothing has caused a significant decrease in the QOL mainly due to the impact on the social component. Thus, change in the style of dressing may be a factor which limits their social interactions and in turn it affects the QOL. Furthermore, other researches also showed similar findings, although did not specifically mention a statistically significant difference in QOL among those who were affected by change in style of clothing [9, 14, 15]. Therefore special attention should be given when ostomy siting is done pre-operatively so that change in style of clothing is minimized.

About 46% “felt depressed” soon after surgery which could be considered as a considerable proportion. The QOL was significantly low in those who “felt depressed” soon after surgery even after a median follow up duration of 38 months. Furthermore the QOL was significantly low in both overall score and scores on social relationships in those who had suicidal thoughts. Thus poor psychological well-being has a significant negative impact on the QOL especially on the social component. This finding is consistent with similar studies [16, 17]. This highlights the importance of psychological interventions in ostomy patients. The patients with depressive thoughts soon after surgery should be counselled appropriately and closely followed up to implement timely interventions.

There was a statistically significant positive correlation between self-efficacy in stoma care and the QOL similar to other studies [18, 19]. However this should be interpreted cautiously as the score measures the perception of self-efficacy and not the actual self-efficacy. More than 50% of the participant scored above 75 which indicate high perception of self-efficacy. Furthermore there was a statistically significant negative association between time taken to learn to care for ostomy and QOL which implies sooner the learning in the initial period after surgery, the higher the long term QOL. This reflects the importance of education and training on stoma care in the initial period to improve QOL. Thus, education and training on ostomy care prior to surgery, and routine assessment of the skills of ostomy care during follow up visits by a specialised stoma therapist would be ideal.

We also acknowledge certain limitations in this study. As the study population is small and also they were treated at a single unit, the results cannot be generalized. Since it is a cross sectional analysis the causal inferences cannot be made in this study.

## Conclusions

To our knowledge this is the first study in Sri Lanka which assessed the QOL using validated instruments and analysed the possible contributory factors. Even though the study is limited by its small sample size, important observations were made in this study. The nature of problems faced by patients in developing countries in South Asia is different due to the difference in the cultural and ethnic background.

We noticed a significantly low QOL in ostomy patients. However we recommend large scale multicentre research to gain further insight and knowledge in the local setting. Furthermore areas like psychological well-being and sexual problems need to be studied as there is scarcity of insight in this aspect among Sri Lankan population. Health care providers should also address the multi-dimensional problems which include the psychosocial and sexual aspects in addition to the physical illness. Recruiting specialized stoma therapists and integrating with other non-surgical specialties may help to achieve this.

## Abbreviations

QOL: quality of life
SQOL: stoma quality of life
SCSE: stoma care self-efficacy scale
BMI: body mass index
